# Structural Analysis and Stochastic Modelling Suggest a Mechanism for Calmodulin Trapping by CaMKII

**DOI:** 10.1371/journal.pone.0029406

**Published:** 2012-01-18

**Authors:** Melanie I. Stefan, David P. Marshall, Nicolas Le Novère

**Affiliations:** EMBL-European Bioinformatics Institute, Hinxton, UK; Michigan State University, United States of America

## Abstract

Activation of CaMKII by calmodulin and the subsequent maintenance of constitutive activity through autophosphorylation at threonine residue 286 (Thr286) are thought to play a major role in synaptic plasticity. One of the effects of autophosphorylation at Thr286 is to increase the apparent affinity of CaMKII for calmodulin, a phenomenon known as “calmodulin trapping”. It has previously been suggested that two binding sites for calmodulin exist on CaMKII, with high and low affinities, respectively. We built structural models of calmodulin bound to both of these sites. Molecular dynamics simulation showed that while binding of calmodulin to the supposed low-affinity binding site on CaMKII is compatible with closing (and hence, inactivation) of the kinase, and could even favour it, binding to the high-affinity site is not. Stochastic simulations of a biochemical model showed that the existence of two such binding sites, one of them accessible only in the active, open conformation, would be sufficient to explain calmodulin trapping by CaMKII. We can explain the effect of CaMKII autophosphorylation at Thr286 on calmodulin trapping: It stabilises the active state and therefore makes the high-affinity binding site accessible. Crucially, a model with only one binding site where calmodulin binding and CaMKII inactivation are strictly mutually exclusive cannot reproduce calmodulin trapping. One of the predictions of our study is that calmodulin binding in itself is not sufficient for CaMKII activation, although high-affinity binding of calmodulin is.

## Introduction

Calcium/calmodulin-dependent kinase II (CaMKII) [1], a highly abundant neuronal protein, has been implicated in learning and memory. Knockout mice that cannot express the 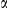 isoform of CaMKII show deficiencies in spatial learning [2] and also in hippocampal long-term potentiation (LTP) [3]. Long-term potentiation is an activity-dependent increase in synaptic strength [4] that has long been associated with learning and memory [4], [5]. On a molecular level, the coincident activity of a pair of neurons triggers a calcium signalling cascade that will lead to a strengthening of the synaptic connection: Upon activation of AMPA receptors by glutamate, the postsynaptic neuron is depolarised, relieving the Mg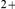 block that inhibits NMDA receptor function under basal conditions [6], [7]. The resulting calcium influx through the NMDA receptor leads to the activation of CaMKII via calmodulin [8]–[10]. Active CaMKII enhances the function of AMPA receptor channels by phosphorylating their GluR1 subunit [11]. It also mediates an increase of AMPA receptor delivery to the postsynaptic membrane [12]. The role of CaMKII in postsynaptic calcium signalling and its abundance in neurons support the view that CaMKII is a key protein in LTP induction and learning.

The CaMKII holoenzyme is dodecameric, organised as a hexamer of dimers [13] with the appearance of two stacked rings [14]. Each subunit can adopt two distinct conformations: An autoinhibited “closed” conformation, in which the active site is bound to the auto-inhibitory helix [13], and an active “open” conformation, in which this interaction is disrupted. Calmodulin stabilises CaMKII activity by binding to the inhibitory helix [15]. The open state of CaMKII is further stabilised by auto-phosphorylation at threonine residue 286 (Thr286) [16], which confers calmodulin-independent activity [17]. Phosphorylation at Thr286 increases the apparent affinity of CaMKII for calmodulin [18]. This is due to a decrease in the rate at which calmodulin dissociates. A mechanistic explanation for this phenomenon, called “calmodulin trapping”, is yet to be found. It has been suggested, however, that the phenomenon of calmodulin trapping might be related to the existence of two calmodulin binding sites on each CaMKII subunit: one high-affinity binding site within residues 291–312 and one low-affinity binding site within residues 298–312 [19].

In order to better understand the molecular basis of calmodulin trapping, we used a combination of structural modelling and stochastic simulations of CaMKII regulation. We show here that calmodulin binding in itself is not sufficient for CaMKII activation, and that calmodulin trapping can be explained by the existence of two binding sites.

Several models of various aspects of CaMKII function exist. Some of these have not included calmodulin trapping at all, because they were concerned with other aspects of CaMKII function, such as frequency dependence [20] or bistability [21]–[23]. Models that did include calmodulin trapping (e.g. [24]–[27]) have modelled it explicitly, as an ad hoc change in calmodulin affinity once a CaMKII subunit is phosphorylated. Our model is the first one to offer a mechanistic explanation of calmodulin trapping.

## Results

To investigate whether binding of calmodulin to the presumed high-affinity binding site was structurally plausible and to investigate the effect that this would have on the CaMKII subunit, we turned to structural modelling.

### Closed and open conformations explored in the absence of Calmodulin

**Figure 1:**
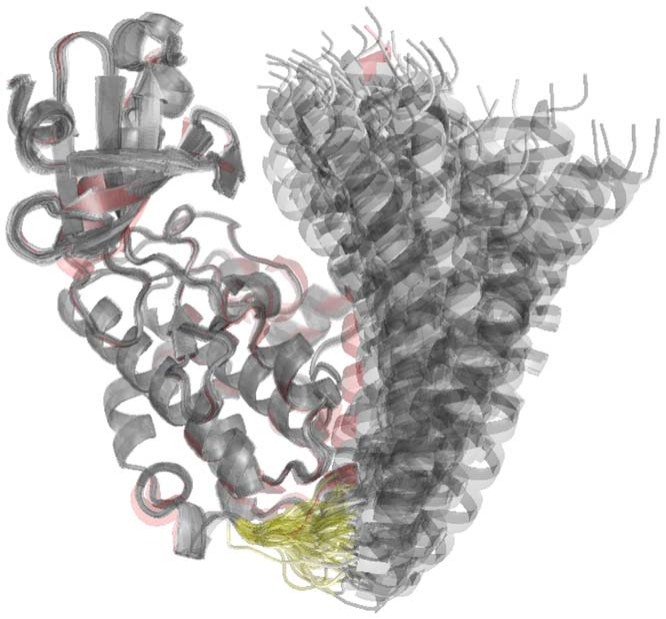
Opening of a CaMKII subunit. Overlay of 100 model structures created with MODELLER, where structural information was omitted for the four residues linking the kinase domain of CaMKII with the inhibitory helix. These four residues are shown in yellow. The structure corresponding to the published structure of the kinase domain (with the linker region intact, PDB ID: 2BDW, chain A) [13] is shown in red.

**Figure 2:**
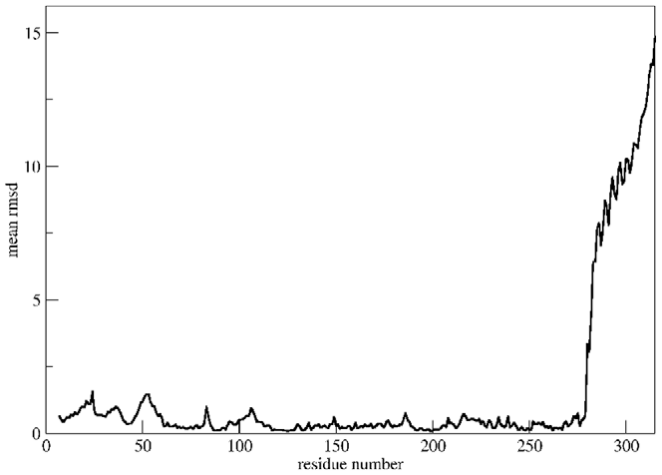
Opening of a CaMKII subunit: Mean RMSD per residue. Average root-mean-square deviation (RMSD) per residue for the structures shown in figure 1. RMSD values were computed using Chimera [53].

We first investigated the range of conformations a single CaMKII subunit can explore if it is allowed to open up, i.e. if the linking region between the inhibitory helix and the rest of the kinase domain is allowed some flexibility. We used a previously published structure of the CaMKII kinase domain from *C. elegans* CaMKII (PDB ID: 2BDW, chain A) [13] and performed structural modelling where information about the four residues that link the inhibitory helix to the active domain were left out. An overlay of 100 structures is shown in Figure 1, and average root-mean-square deviation (RMSD) for each residue is plotted in Figure 2. In the absence of further constraints, the flexible linker allows for considerable movement of the inhibitory helix with respect to the kinase domain, ranging from structures which are essentially closed, i. e. where the catalytic domain is masked by the auto-inhibitory helix, to open structures, where the kinase domain is accessible. This is consistent with a recent study by Hoffman et al. who found that in the absence of calmodulin, CaMKII exists as a conformeric equilibrium between structures where the autoinhibitory helix and the kinase domain interact and structures where this interaction is disrupted [28].

### Low-affinity binding of calmodulin does not interfere with the closed conformation of CaMKII

**Figure 3:**
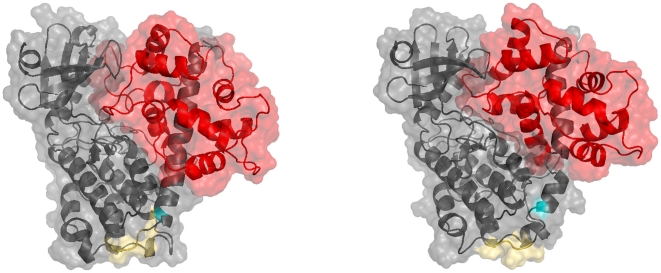
Models of Calmodulin bound to the low-affinity site of a CaMKII subunit. Left panel: Binding to a CaMKII subunit in a closed conformation. Right panel: Binding to a CaMKII subunit with flexible linker. Although the linker region between inhibitory helix and catalytic domain was flexible, the complex CaMKII-calmodulin still favoured a closed conformation of CaMKII. Calmodulin is shown in red, CaMKII in grey. The Thr286 autophosphorylation site is shown in teal, the region that links the catalytic domain to the autoinhibitory helix in yellow.

To model the structure of calmodulin bound to the low-affinity binding site of CaMKII, we used previously published structures of the kinase domain of CaMKII (PDB ID: 2BDW, chain A) [13] and of calmodulin bound to a fragment of the inhibitory helix of CaMKII (PDB ID: 1CM1, chains A and B) [29] for structural modelling and molecular dynamics simulations. The structural model suggests that calmodulin binding to the low-affinity binding site of CaMKII is compatible with closure of the CaMKII subunit (see Figure 3, left panel). A PDB file of the resulting structure can be found in Dataset S1.

**Figure 4:**
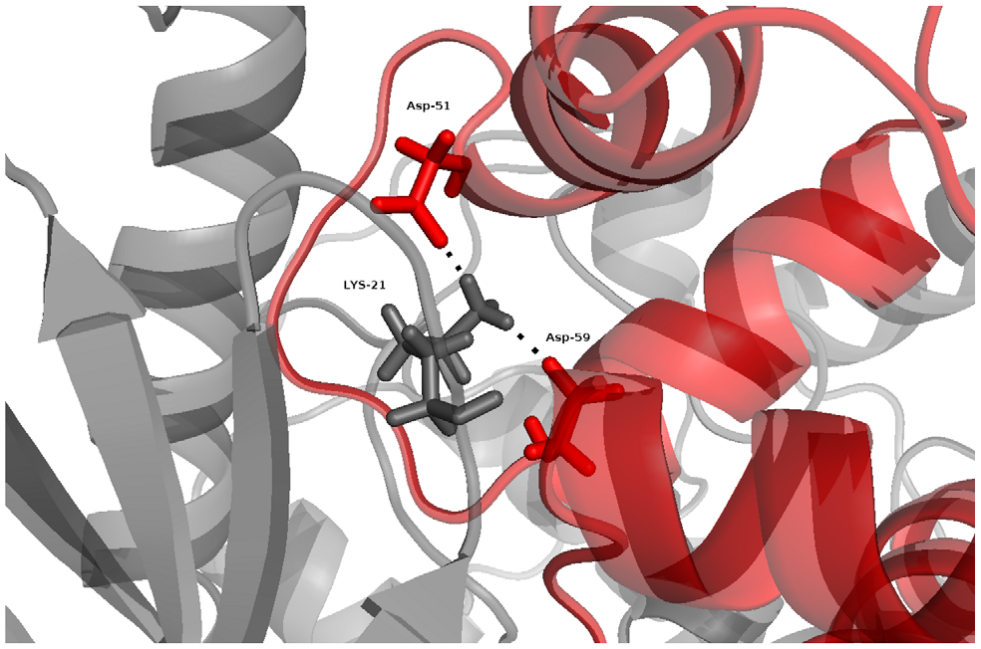
Interactions between calmodulin and its low-affinity binding site on the kinase domain of CaMKII. Interacting residues are shown as sticks. Calmodulin is shown in red and CaMKII in grey.

To test whether low-affinity binding of calmodulin to CaMKII might favour a more open structure, we gave the inhibitory helix of CaMKII freedom to move by introducing a flexible linker between the helix and the kinase domain. The model suggests that even if the inhibitory helix is given freedom to move away from the catalytic domain, the closed form is preferred (see Figure 3, right panel). This may be due to an interaction between residues Asp51 and Asp59 in the calmodulin structure (PDB ID: 1CM1, chain A) and Lys20 (in *C. elegans*, corresponding to Lys21 in mouse or rat) in the catalytic domain of CaMKII (PDB ID: 2BDW, chain A). The interaction is highlighted in Figure 4. A similar interaction between calmodulin and the catalytic kinase domain has recently been reported for calmodulin binding to death-associated protein kinase (DAPK) [30]. In the absence of this interaction, low-affinity binding of calmodulin would be compatible with further opening of the CaMKII subunit. This would be the case, for instance, when CaMKII is locked in the open conformation by autophosphorylation at Thr286. A structure of calmodulin bound to an open conformation of CaMKII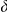 has indeed been observed (PDB ID: 2WEL) [31].

Thus, the structural model suggests that calmodulin binding to the low-affinity binding site on CaMKII can happen independently of whether the CaMKII subunit is open or closed. This would also mean that binding of calmodulin does not necessarily stabilise the active conformation of CaMKII, at least as long as calmodulin is bound to the low-affinity site.

### High-affinity binding of calmodulin requires opening of CaMKII

In order to model calmodulin binding to the high-affinity binding site on CaMKII, we modified the structure of calmodulin bound to a fragment of the inhibitory helix of CaMKII (PDB ID: 1CM1, chains A and B) [29] by manually shifting the position of calmodulin by one turn of the helix, corresponding to the supposed position of the high-affinity binding site. We then used this structure and the structure of the kinase domain of CaMKII (PDB ID: 2BDW, chain A) [13] to create a combined structural model. This approach, however, failed to give a valid structure without overlaps. We concluded that calmodulin binding to the proposed high-affinity binding site is impossible if CaMKII is in the closed conformation.

**Figure 5:**
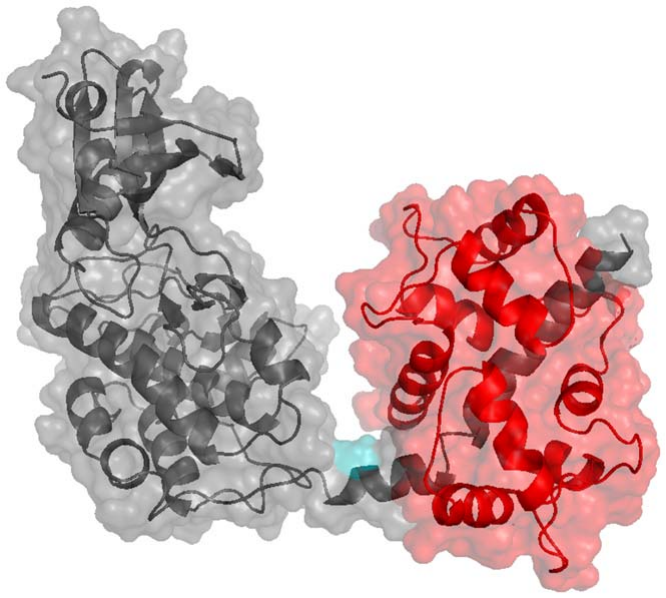
High-affinity binding of calmodulin to the open state of CaMKII. Calmodulin is shown in red, CaMKII in grey and the autophosphorylation site at Thr286 in teal.

To further test this hypothesis, we repeated our modelling approach, this time omitting structural information pertaining to the linker region between the inhibitory helix and the kinase domain, so that the two domains were free to move with respect to each other. Under these conditions we could indeed obtain an overlap-free structure of the calmodulin-CaMKII complex (see Figure 5). A PDB file of this structure is provided in Dataset S2.

It is interesting to note that this structure requires a considerable degree of opening of the CaMKII subunit, thereby exposing the autophosphorylation site at Thr286. Unlike in the model of calmodulin binding to the low-affinity site, there seems to be no interaction between calmodulin and the catalytic domain of CaMKII, which allows CaMKII to adopt an open state and facilitates substrate access to the catalytic site. In addition, calmodulin binding to the high-affinity site seems to cause some degree of local conformational change in the inhibitory helix.

Together, these results suggest that binding of calmodulin to the low-affinity site is independent on whether CaMKII is open or closed, but that binding to the high-affinity site requires opening of CaMKII, and will therefore stabilise the active open state.

### High-affinity binding includes residues crucial for calmodulin trapping

**Figure 6:**
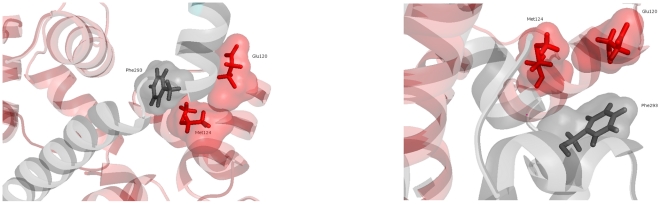
Residues crucial for calmodulin trapping. Left panel: In the high-affinity structure, residue Met124 on calmodulin (in red) makes contact both with residue Glu120, also on calmodulin and with residue Phe293 on CaMKII (in grey). Right panel: In the low-affinity structure, these contacts are missing.

Having obtained a structural model of calmodulin binding to an open state of CaMKII, we examined whether this structure could be relevant for calmodulin trapping. By using site-directed mutagenesis, Singla et al. [32] have shown residues Phe293 on CaMKII and Glu120 and Met124 on calmodulin to be crucial for calmodulin trapping by CaMKII. Indeed, in the high-affinity structure, Met124 on calmodulin makes contact with both Phe293 on CaMKII and Glu120 on calmodulin (see Figure 6, left panel), a feature not found in the low-affinity structure (see Figure 6, right panel).

This suggests that binding of calmodulin to the high-affinity site on CaMKII may play an important role in calmodulin trapping.

### Only one calmodulin binding site can be occupied at any given time

**Figure 7:**
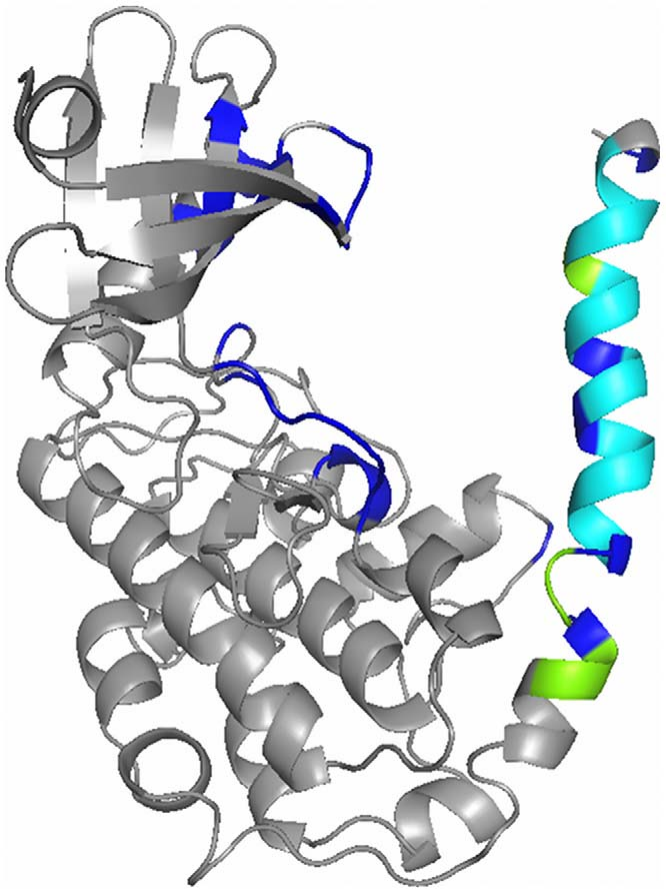
Interaction surfaces for low-affinity and high-affinity binding. The interaction surfaces for calmodulin binding are projected onto a CaMKII monomer. The low-affinity interaction surface is shown in blue, the high-affinity interaction surface in green, and the overlap in cyan. Interaction surfaces were computed using Chimera [53] with a 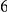
Å cutoff.

The structural model also suggests that, although there might well be two binding sites for calmodulin on each CaMKII subunit, no more than one calmodulin molecule can be bound at any given time. Figure 7 shows that there is considerable overlap between the two binding sites. This means that the actual stoichiometry of CaMKII binding to calmodulin is still at most one calmodulin bound per subunit of CaMKII. Due to the proximity of the two binding sites, however, calmodulin binding to the low-affinity site will greatly increase the effective local concentration of calmodulin around the high-affinity binding site, and vice versa. Thus, a calmodulin molecule could effectively stay associated with a CaMKII subunit, while “sliding” back and forth between the low-affinity and the high-affinity binding site.

Thus, the two binding sites for calmodulin on CaMKII do not allow more than one calmodulin molecule to bind at any given time, but provide two modes of binding to CaMKII for a single calmodulin molecule.

### The structural model is compatible with a two-binding-sites hypothesis

Taken together, the results suggest that the structural model of calmodulin binding to CaMKII is indeed compatible with the existence of two calmodulin binding sites on CaMKII, as suggested by Tse et al. [19]. Binding to one of these sites (the low-affinity site) would be compatible with closure and hence, inactivity of the CaMKII subunit, while binding to the other (high-affinity) site would require CaMKII to be open. The presumed high-affinity binding event involves residues previously implied in calmodulin trapping [32]. To test whether this two-binding-sites model could explain calmodulin trapping by autophosphorylated CaMKII, we set up a stochastic model of these interactions.

### Stochastic simulations of calmodulin trapping by CaMKII

Using the results from structural modelling, we have designed a rule-based model of calmodulin trapping by CaMKII. In this model, CaMKII is represented as a hexamer. This is a compromise between modelling CaMKII as a collection of monomeric subunits and modelling CaMKII as a dodecameric holoenzyme. A model in which CaMKII is represented as unconnected monomeric subunits would be sufficient in order to study the core mechanisms for calmodulin trapping (including the two binding sites for calmodulin, opening and closing of the subunit and the effect of Thr286 phosphorylation on calmodulin affinity). However, it would make it harder to accurately describe Thr286 autophosphorylation, which is a neighbour-sensitive reaction between adjacent subunits on the same hexameric ring. On the other hand, representing CaMKII as a full dodecamer would make it necessary to include other kinds of neighbour-sensitive interactions - many of them as yet poorly understood - but would not provide any additional insights into the trapping mechanism.

In our model, each subunit of CaMKII can be open or closed, phosphorylated at threonine residues 286 and 306 and bound to calmodulin on either the high-affinity or the low-affinity binding site. The open form is assumed to be catalytically active. This results in a model where each subunit has five binary state flags: activity, phosphorylation at Thr286, phosphorylation at Thr306, binding of calmodulin to either of the two sites and binding of calmodulin to the high-affinity site. (The last two flags are set to 00 if no calmodulin is bound, 10 if calmodulin is bound to the low-affinity site and 11 if calmodulin is bound to the high-affinity site. The combination 01 is impossible.) Autophosphorylation at Thr286 is modelled as a neighbour-sensitive reaction, which can only occur if both the subunit acting as the kinase and the subunit acting as the substrate for the phosphorylation reaction are open. Phosphorylation at Thr286 locks a subunit in the open state. In our model, the corresponding dephosphorylation is mediated protein phosphatase 1 (PP1). Phosphorylation at Thr306 is an intra-subunit autophosphorylation and therefore depends on the subunit in question being active. Phosphorylation at this residue, however, does not interfere with closing of the subunit. Since both the presumed high-affinity and low-affinity binding domains for calmodulin contain residue Thr306, phosphorylation at this residue and calmodulin binding are modelled to be mutually exclusive. Following the results of the structural model presented above, calmodulin binding to the high-affinity binding site precludes closing of a CaMKII subunit, whereas binding to the low-affinity site does not interfere with closing.

**Figure 8:**
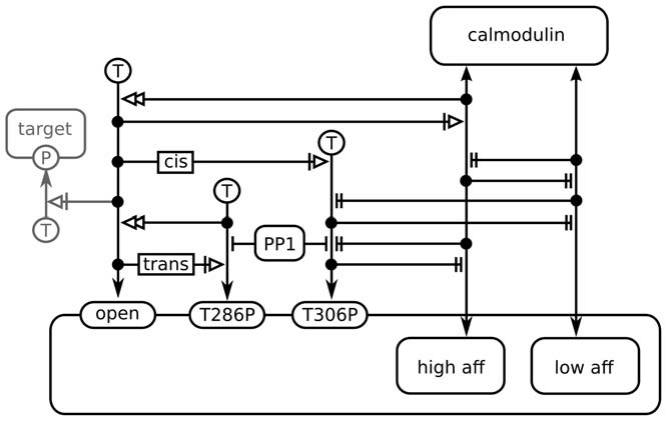
Model of calmodulin trapping by CaMKII. The model is shown as an SBGN ER diagram [54]. For clarity, only one monomeric subunit is shown. In the actual model, six such subunits form a ring, and autophosphorylation at Thr286 of one subunit is dependent on the neighbouring subunit being open.

A diagram of the reaction scheme used in the stochastic model can be found in Figure 8. A full list of reaction rules is given in table 1.

**Table 1:** List of reactions for the model of calmodulin trapping by CaMKII.

Phosphorylation of CaMKII at Thr286	Substrate: CaMKIIProduct: CaMKIISets flag on neighbour: +P286Needs flag: +openNeeds flags on neighbour: +open −P286
Dephosphorylation of CaMKII at Thr286 by PP1	Substrates: CaMKII, PP1Products: CaMKII, PP1Sets flag: −P286Needs flag: +P286
Phosphorylation of CaMKII at Thr306	Substrate: CaMKIIProduct: CaMKIISets flag: +P306Needs flags: +open −P306 –calm
Dephosphorylation of CaMKII at Thr306 by PP1	Substrates: CaMKII, PP1Products: CaMKII, PP1Sets flag: −P306Needs flag: +P306
Calmodulin binding to CaMKII (low-affinity site)	Substrates:CaMKII, calmodulinProduct: CaMKIISets flag: +calmNeeds flags: −P306 -calm –ha
Calmodulin dissociating from CaMKII (low-affinity site)	Substrate: CaMKIIProducts: CaMKII, calmodulinSets flag: −calmNeeds flags: +calm –ha
Calmodulin binding to CaMKII (high-affinity site)	Substrates: CaMKII, calmodulinProduct: CaMKIISets flags: +calm +haNeeds flags: +open −P306 −calm −ha
Calmodulin dissociating from CaMKII (high-affinity site)	Substrate: CaMKIIProducts: CaMKII, calmodulinSets flags: −calm −haNeeds flags: +calm +ha
Opening of CaMKII (rapid equilibrium)	Substrate: CaMKIIProduct: CaMKIISets flag: +openProbability: 1 if +P286 or +ha, 0.004 else
“Sliding” of calmodulin to the high-affinity site(rapid equilibrium)	Substrate: CaMKIIProduct: CaMKIISets flag: +haProbability: 0.99997 if +open and +calm

List of reactions for the model of calmodulin trapping by CaMKII.

### The two-binding-sites model can reproduce trapping

**Figure 9:**
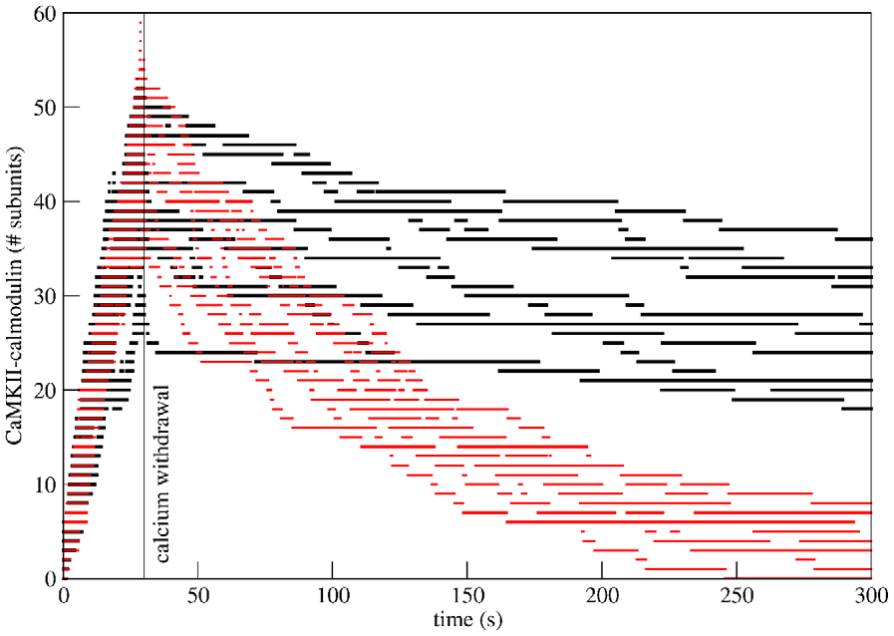
Several trapping simulations on wildtype and mutant CaMKII. Calmodulin is inactivated, mimicking calcium withdrawal after 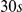. The ratio of calmodulin to CaMKII concentration used in the simulation was the same as used in the experimental setup by Meyer et al. [18]. The number of calmodulin-bound monomeric CaMKII subunits is plotted against time for each simulation run. The total number of CaMKII subunits in the simulation was 360. Wildtype is shown in black, T286A mutant in red. Ten simulation runs are shown for each.

To assess whether our model can reproduce the trapping of calmodulin observed in vitro, we ran stochastic simulations on both wildtype CaMKII and an in silico mutant version that cannot be phosphorylated at Thr286. Following the experimental procedure of Meyer et al. [18], the system was allowed to saturate for thirty seconds, and calmodulin then inactivated, corresponding to the withdrawal of calcium in the experimental setup. The ratio between calmodulin and CaMKII concentration used for this simulation was the same as used by Meyer et al. [18] (60 hexamers of CaMKII for 450 molecules of calmodulin), and no phosphatase was present. To ensure that the observed result is not just a random effect, the same simulation was repeated ten times on wildtype and mutant CaMKII. The simulations (see Figure 9) show that although both versions of CaMKII were equally saturated with calmodulin after thirty seconds, calmodulin dissociation proceeded slower from the wildtype than from the mutant, showing a trapping effect that is, indeed, due to different apparent off rates.

**Figure 10:**
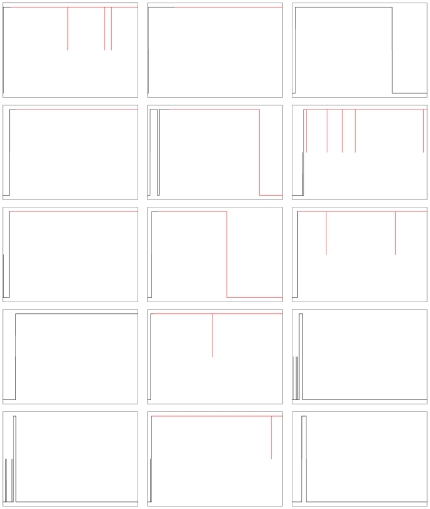
Calmodulin trapping on the level of single subunits. Each individual panel represents a single subunit chosen at random from the simulations from those subunits that bind calmodulin. The x-axis represents time, going from 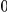 to 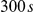. The three levels in the y-dimension represent calmodulin binding, with no binding (lowest level), low-affinity binding (middle level) and high-affinity binding (highest level). Events of calmodulin sliding back and forth between the high-affinity and the low-affinity binding sites appear as drops from the top level to the centre and back up. The colour of the trace represents subunit phosphorylation at Thr286, with unphosphorylated subunits shown in black, and phosphorylated subunits in red.

Figure 10 shows examples of calmodulin binding behaviour at the level of the individual subunit. From one of the ten simulation runs above, we chose fifteen subunits at random out of all subunit that undergo calmodulin binding during the course of the simulation and plotted calmodulin binding and phosphorylation over time. The Figure shows that calmodulin binding behaviour varies widely across subunits. Note that since calmodulin was inactivated after 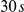, no new binding events are seen from then on, so whether calmodulin is bound to a subunit depends entirely on dissociation. While direct dissociation from the high-affinity site does occur, movement of calmodulin from the high-affinity site to the low-affinity site and back again is more frequent, especially in phosphorylated subunits.

Taken together, this shows that the two-binding-sites model presented here can reproduce calmodulin trapping without having to postulate changes in microscopic association or dissociation parameters upon phosphorylation of CaMKII. The existence of these two binding sites for calmodulin is thus sufficient to explain calmodulin trapping.

### A one-binding-site model cannot reproduce trapping

Could calmodulin trapping be explained by an alternative model? Our model relies on two key assumptions: First, that each CaMKII subunit has two calmodulin binding sites and second, that binding to the low-affinity binding site does not necessarily entail CaMKII opening and thus, activation. This naturally raises the question of whether a model that does not rely on these two assumptions could also reproduce calmodulin trapping.

**Figure 11:**
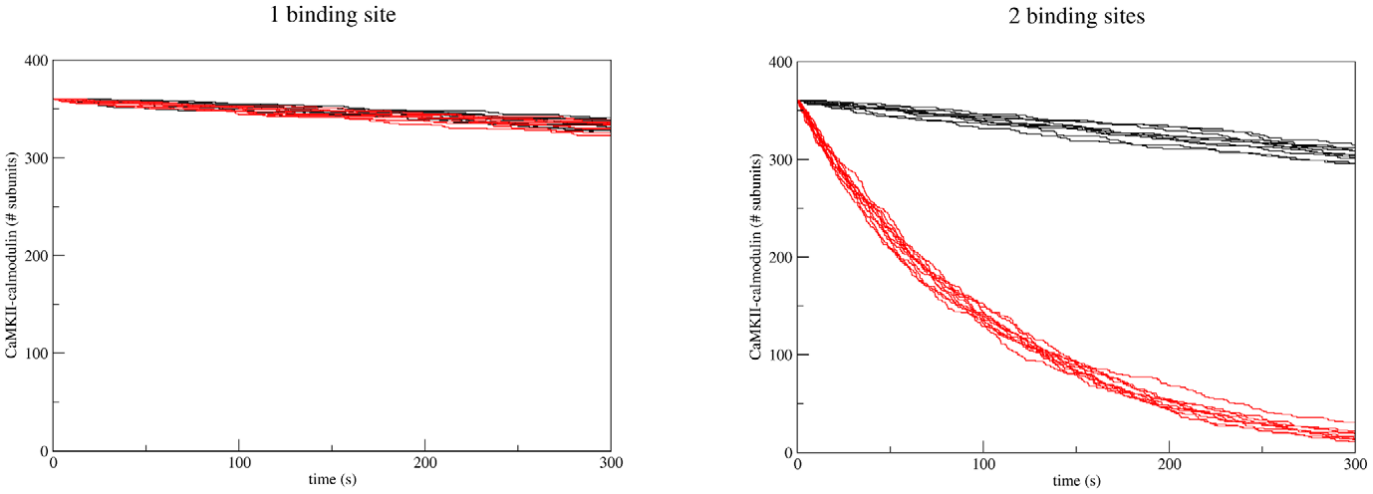
Comparison of the two-binding-site model with a one-binding site model. For each of the models, we ran ten simulations with wildtype CaMKII and ten simulations with the autophosphorylation deficient T286A mutant. All CaMKII molecules are open and fully saturated with calmodulin to begin with, and calmodulin is withdrawn, mimicking calcium withdrawal, at the start of the simulation. The ratio of calmodulin to CaMKII concentrations was the same as used in the experimental setup by Meyer et al. [18]. The number of calmodulin-bound monomeric CaMKII subunits is plotted against time. Wildtype is shown in black, T286A mutant in red. There is no difference in slope between mutant and wildtype in the one-binding-site model, whereas the two-binding-site model displays a clear difference between wildtype and mutant.

In order to address this issue, we constructed a model of CaMKII with only one (high-affinity) calmodulin binding site. In this model, calmodulin binding and closing of a CaMKII subunit are mutually exclusive, meaning that calmodulin binding is sufficient for CaMKII activation. All other reactions and parameters are the same as in the model presented above. Figure 11 (left panel) shows the pooled results of ten simulations on wildtype CaMKII and on Thr286-to-alanine (T286A) mutant CaMKII with this alternative model. As in the trapping simulation presented above, CaMKII was first saturated with calmodulin (here: at the beginning of the simulation in order to separate the effect of Thr286 on calmodulin binding from its effect on calmodulin dissociation) and all free calmodulin then withdrawn, such that dissociation of calmodulin from CaMKII could be monitored. The results show that this alternative model cannot reproduce the change in apparent 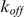 that characterises calmodulin trapping. For comparison, the same simulation was run with our two-binding-site model (Figure 11, right panel), showing a clear difference between wildtype and mutant CaMKII.

Taken together, these results suggest that the existence of two binding sites for calmodulin on a single CaMKII subunit is not only sufficient, but might indeed be necessary for trapping. If only one binding site is used in vivo, then another, yet unknown mechanism must be in place to enable calmodulin trapping.

## Discussion

### Assumptions underlying our model

The model presented above makes assumptions regarding both the choice of initial conditions for simulations and the fundamental mechanisms involved in calmodulin binding to CaMKII. Initial conditions were chosen to match the conditions used in the experimental paper that first reported calmodulin trapping [18]. This is why calmodulin concentration is saturating, the entire population of calmodulin molecules is assumed to be active at the beginning of the simulation and CaMKII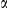 is the only isoform considered. This reflects the situation in the test tube, and therefore, like an in vitro experiment, allows us to isolate and explain the particular mechanism of calmodulin trapping. This is also why the unit of analysis in our model is the hexameric ring, because, given our current knowledge, this is best suited to study calmodulin trapping: It includes the neighbour-sensitivity of Thr286 autophosphorylation, but disregards possibly confounding and as yet ill-understood effects of dodecameric assembly. In order to understand the role that calmodulin trapping plays in postsynaptic signalling and, ultimately, synaptic plasticity, it will be necessary to place the model in a context that better mimics in vivo conditions. This includes a more detailed representation of calmodulin activation, CaMKII topology and subunit composition, accurate calcium dynamics, the presence of other proteins and spine geometry.

Regarding the binding mechanisms, our model makes two important assumptions: First, that there are two binding sites for calmodulin on CaMKII and second, that binding to one of these sites is compatible with closing of CaMKII, i. e. that calmodulin binding is not sufficient for CaMKII activation. By measuring calmodulin binding to CaMKII peptides of different lengths, Waxham et al. [33] and Tse et al. [19] have made a plausible case for the first assumption, though whether both binding sites are actually used for calmodulin binding to full-length CaMKII in vivo has not been experimentally confirmed so far. (Note, however, that experimental work by Chin and Means [34] on full-length CaMKII seems to be consistent with the existence of two calmodulin binding sites, although the authors themselves do not draw the same conclusion). The second assumption is somewhat more controversial; in fact, most of the literature on CaMKII implicitly or explicitly assumes that calmodulin binding is sufficient for CaMKII activation (reviewed in [35]). The question therefore arises whether both these assumptions are necessary in order to reproduce trapping of calmodulin by CaMKII.

We therefore developed a corresponding one-binding-site model, where calmodulin binding is incompatible with CaMKII closing and therefore sufficient for CaMKII activation. This model has only one (high-affinity) binding site, and the apparent CaMKII affinity for calmodulin is slightly higher than for the wildtype in the two-binding-site model. This is because there is no exchange and no competition between high-affinity and low-affinity binding sites in the one-binding-site model, such that all calmodulin binding is concentrated on the high-affinity binding site. Crucially, this model could not reproduce calmodulin trapping: The apparent 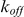 of calmodulin is the same for wildtype and T286A mutant CaMKII. This suggests that at least one of the two conditions needs to be fulfilled in order for calmodulin trapping to happen: the existence of two binding sites or the compatibility of calmodulin binding and CaMKII inactivation.

### Failure of a one-binding-site model to reproduce calmodulin trapping

The failure of the alternative one-binding site model to reproduce calmodulin trapping is perhaps not surprising: In the wildtype, the higher number of open subunits due to Thr286 autophosphorylation will increase the apparent 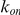 for calmodulin binding (since more binding sites will be available), but this has no influence on the apparent 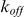 after calcium withdrawal. Consider what happens to a calmodulin molecule bound to a CaMKII subunit in a one-binding-site scenario. Once calmodulin has dissociated from the only binding site, it is very quickly inactivated due to the lack of calcium, and is therefore no longer available to bind again. Importantly, this is independent of the autophosphorylation state of the subunit from which calmodulin has dissociated, so there is no difference in apparent 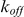 between wildtype and T286A autophosphorylation mutants. In order to reproduce trapping, a one-binding-site model would need to include an ad hoc increase in calmodulin affinity for autophosphorylated CaMKII, as has indeed been done in previous models of CaMKII activation (e.g. [24]–[27]). This will reproduce the effect, but without providing an explanation of the mechanism.

The full trapping model presented here overcomes the need for an ad hoc increase in affinity by postulating the existence of an additional binding site. In this case, not all of the calmodulin dissociating from the high-affinity site is immediately inactivated upon calcium withdrawal, but some of it merely “slides” to the low-affinity binding site and thus remains on the same subunit. In this case, autophosphorylation does matter: Phosphorylated subunits remain open and calmodulin can therefore “slide back” to the high-affinity binding site. In contrast, unphosphorylated subunits are likely to close, which makes re-binding to the high-affinity site impossible. The existence of a second binding site is thus important in order to retain calmodulin in the vicinity of the high-affinity binding site for a while after it has dissociated, rather than releasing it completely.

By the same argument, binding to one of the binding sites has to be compatible with closing of the CaMKII subunit. Otherwise, a CaMKII subunit would only be able to close once calmodulin has completely dissociated from it, which means that the additional stabilisation of the open state by autophosphorylation at Thr286 has no effect on the apparent 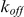 (although, again, it would have an effect on apparent 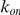). Thus, a two-binding-sites model where binding of calmodulin to one of the sites is compatible with the CaMKII subunit closing seems to be necessary for calmodulin trapping, unless some other yet unknown mechanism is involved.

It has been suggested that autophosphorylation at Thr286 could induce a conformational change which increases calmodulin affinity [28]. Interestingly, a key residue involved in this conformational change, residue Phe293 [28], [32] is specific to high-affinity calmodulin binding in our model (see Figure 7). Our view is that phosphorylation at Thr286 stabilises a conformation conducive to calmodul in binding (rather than inducing such a conformation), but this interpretation is, as far as we can see, compatible with the structural data [28].

To summarise, the existence of two modes of calmodulin binding to CaMKII seems to be compatible with a wealth of existing experimental data. We show that this is both necessary and sufficient to explain calmodulin trapping and provide the first computational model in which calmodulin trapping arises as a feature of the entire system, rather than being hard coded as an explicit change in macroscopic parameters.

### Binding of calmodulin to a closed structure of CaMKII

As described above, the trapping model proposed here requires calmodulin being able to bind to closed forms of CaMKII, a requirement which seems structurally plausible. This means that binding of calmodulin to CaMKII as such is not sufficient for CaMKII activation, although binding of calmodulin to the high-affinity site is. But the structural model goes even further than that: Not only does calmodulin binding to the low-affinity site seem compatible with closing, but it even seems to favour the closed conformation of CaMKII. This is because calmodulin, while bound to the inhibitory helix, also interacts with a lysine residue in the catalytic domain of CaMKII, thereby keeping those two domains in close proximity. This is a key prediction of our model, and one that has not been made elsewhere. Further investigations will be necessary to shed light on the detailed mechanism and functional role of such an interaction.

### Detailed mechanism of calmodulin binding to CaMKII

Calmodulin has two calcium-binding lobes which are connected by a flexible helix. Both lobes are involved in binding CaMKII. We have previously published an allosteric model describing calcium binding to calmodulin and the calcium-dependent activation of CaMKII [36]. It has been suggested, however, that calmodulin binding to CaMKII is a sequential process where one lobe of calmodulin makes contact with CaMKII first, which then facilitates binding of the second lobe [37]–[39]. Such a mechanism would allow for a low-affinity binding mode (when only one lobe of calmodulin is bound to CaMKII) and a high-affinity binding mode (when both lobes are bound) to exist even in the absence of two calmodulin binding sites. This raises the possibility that what has been identified as a low-affinity and a high-affinity binding site in the experiments by [19] could actually just reflect partial vs full binding of calmodulin to CaMKII. However, the published structure of calmodulin binding to what corresponds to the low-affinity binding site on the CaMKII helix (PDB ID: 1CM1, chains A and B) [29] is one of fully bound calmodulin, suggesting that two calmodulin binding sites do indeed exist.

Our model has disregarded partial binding of calmodulin. The first reason for this is the design of our structural model, which was based on a starting structure of calmodulin fully bound to CaMKII (PDB ID: 1CM1, chains A and B) [29], i.e. where calmodulin “wraps around” its target. Our molecular dynamics simulations have not allowed for flexibility within the calmodulin molecule, thus preventing us from picking up hypothetical structures of calmodulin that is partly bound to CaMKII (and where, presumably, the conformation of the flexible helix connecting the two lobes would be quite different from the fully bound state). Second, we consider partial binding of only the N- or the C-lobe of calmodulin to CaMKII as a transient initial state, which facilitates (and therefore very often results in) full binding of both lobes.

Our model includes calmodulin “sliding” back and forth between the low-affinity and the high-affinity binding sites. These reactions are short-hands for a process or a combination of processes which are not known in detail. For instance, it seems reasonable to assume that since both binding sites are in close proximity to each other, once a calmodulin molecule dissociates from one of the sites, this drastically increases the local calmodulin concentration around the other binding site, and thus the probability of binding there. Similar mechanisms have been well described for facilitated diffusion of proteins along a DNA strand [40]. It is also conceivable that there might be some form of conformational change of calmodulin bound to the low-affinity site, which would allow it to move along the helix and bind to the high-affinity site, as suggested by Tse et al. [19] or that a conformational change in the CaMKII inhibitory helix might be involve in the transition [28]. The two sliding reactions in our model are summaries of these (and possibly other) processes without including details about the exact mechanisms involved. As Figure 10 illustrates, calmodulin sliding is indeed an important mechanism in our simulations, by which a subunit can extend the lifetime of its calmodulin-bound state, and calmodulin can change back and forth between the low-affinity and the high-affinity site several times before dissociation completely.

### Potential experimental validation

While the structure of calmodulin bound to the low-affinity binding site of CaMKII is known, the structure of calmodulin bound to the high-affinity binding site is a prediction from our model. The experimental determination of this structure - for instance as a complex of calmodulin with a longer fragment of the CaMKII autoinhibitory helix than in previous studies - could be used to validate this model.

The model of calmodulin trapping predicts that calmodulin binding is not per se sufficient for CaMKII activation. This is another prediction that could be tested experimentally. This could be done using monomeric T286A mutant CaMKII in order to separate the effect of calmodulin binding from that of autophosphorylation and other inter-subunit interactions. In that scenario, by quantifying both calmodulin binding and CaMKII activation, we predict that there would be a small portion of CaMKII molecules that are not active, even though bound to calmodulin. This portion could be increased by additionally disrupting calmodulin binding to the high-affinity (but not the low-affinity) binding site on CaMKII.

### Conclusion

Calmodulin trapping upon CaMKII autophosphorylation might have an important role in synaptic plasticity by fine-tuning both CaMKII activity and calmodulin availability. We have combined structural modelling and stochastic simulations into a model that offers a detailed mechanistic explanation of calmodulin trapping. The model relies on two main ideas: First, the existence of two calmodulin binding sites on a given CaMKII subunit - an assumption backed by biochemical studies and our own structural work - and second, on the compatibility of calmodulin binding to one of these sites with CaMKII inactivity. Crucially, our model does not rely on any ad hoc assumptions about parameter changes after CaMKII autophosphorylation. Instead, the change in apparent 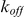 upon autophosphorylation, i.e. calmodulin trapping, is an emergent property that follows from the mechanisms of calmodulin binding and CaMKII conformational change. Our model thus offers a mechanistic explanation of calmodulin trapping, rather than just reproducing the effect. It provides a basis for further research in synaptic plasticity and memory, but possibly also in other fields where CaMKII signalling plays a role, including apoptosis (reviewed in [41], [42]) and the cardiovascular system (reviewed in [43]).

## Methods

### Structure of a CaMKII subunit bound to calmodulin

Structural models were developed using the MODELLER software [44]. In order to obtain the structure of calmodulin-bound CaMKII, we used as templates previously published structures of the kinase domain of *C. elegans* CaMKII (PDB ID: 2BDW, chain A) [13] and of calmodulin bound to a fragment of the inhibitory helix of bovine CaMKII (PDB ID: 1CM1, chains A and B) [29]. The target sequence of calmodulin bound to the full kinase domain of CaMKII was created by combining the sequences of the two template PDB structures (chain A of 2BDW and chain A of 1CM1). The function automodel in MODELLER was used in order to model the unknown structure of the newly generated sequence, using the alignment file and the known structures of 2BDW and 1CM1 as inputs. The initial result featured two interlocking loops (residues 53–61 in the calmodulin structure and residues 159–163 in the kinase domain structure). In order to resolve this issue, loop refinement was performed using the Discrete Optimized Protein Energy (DOPE) method [45] with MODELLER. Out of 10 iterations, the structure with the minimal molpdf score (139.06694) was selected for further refinement using Amber 7 [46]. The ff94 force field was used and the molecule was solvated in a WATBOX216 water box with a 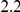 nm cutoff using tleap. We used Sander for energy minimisation (parameters used: imin = 1, maxcyc = 100, cut = 300.0, igb = 2, saltcon = 0.2, gbsa = 1, ntpr = 10, ntx = 1, ntb = 0). The result of the minimisation step was then used as an input for molecular dynamics using sander (parameters used: ntb = 0, ntt = 1, tautp = 0.5, dtemp = 2.0, nstlim = 5000, dt = 0.002, ntc = 2, ntf = 2, cut = 15, nsnb = 9999, ntpr = 20, ntwx = 20, all other parameters set to the default value).

### Modelling CaMKII opening with a flexible helix

In order to model a potential open structure of CaMKII, the structure of the kinase domain (PDB ID: 2BDW, chain A) [13] was split into two parts: the autoinhibitory helix only and the rest of the kinase domain. Structural information about the four residues that link the two domains (Ser277 to His280 in the *C. elegans* sequence) was omitted. This was done in order to allow those residues to move freely in the course of the simulation, thereby allowing the autoinhibitory helix to move away from the kinase domain. Using the automodeller function of MODELLER, we iteratively generated 100 structures using those two partial structures as an input. The same two structures and the structure of calmodulin bound to a fragment of the inhibitory helix (PDB ID: 1CM1, chains A and B) were then used as templates for modelling the structure of calmodulin bound to the low-affinity site of a potentially open state of CaMKII. This was done using MODELLER and loop refinement was again performed using DOPE. Molecular dynamics was performed on the resulting structure using Amber 7: tleap was used for solvation using the ff94 force field and a WATBOX216 water box with a 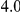 nm cutoff. Sander was used for minimisation (parameters as above) and molecular dynamics (parameters as above).

### Structure of calmodulin bound to the high-affinity site

The fragment containing the high-affinity binding site used by Tse et al. [19] is seven residues longer than the fragment containing the low-affinity binding site. We assumed the high-affinity binding site to be towards the middle of these seven residues, i.e. one turn further towards the inside of the CaMKII inhibitory helix than the low-affinity binding site. Therefore the structure of CaMKII bound to the low-affinity site used before (PDB ID: 1CM1, chains A and B) was modified by manually translating the inhibitory helix by one turn in PyMOL (http://www.pymol.org). Note that this assumption was merely used to find a starting point for simulations away from the established low-affinity binding site. The identification of the precise residues that contribute to the interaction was left to the molecular dynamics simulations. Together with the structure of the kinase domain (PDB ID: 2BDW, chain A), this was then used to create a putative combined structure using the automodel function in MODELLER, as described above. Again, tleap in Amber 7 was used for solvation, this time with a larger solvent box (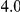 nm). Energy minimisation and molecular dynamics were performed using sander (parameters as described above). Binding of calmodulin to the high-affinity site of a potentially open structure was modelled using separate structures for the inhibitory helix and the rest of the kinase domain, as described above. MODELLER was used to generate an initial structure, which was used as an input for Amber 7: The structure was solvated using tleap (with a 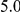 nm water box), and sander was used to run both minimisation and molecular dynamics (parameters as above).

All figures of protein structures in this paper were created using PyMOL [47].

### Kinetics of calmodulin binding to the high-affinity binding site

For biochemical modelling, we needed to determine parameters describing calmodulin binding to the low-affinity binding site of CaMKII, calmodulin binding to the high-affinity binding site and “sliding” of calmodulin from the high-affinity to the low-affinity binding site and back. These latter reactions describe an unknown underlying mechanism (or collection of mechanisms) by which binding of a calmodulin molecule to one of the two binding sites might facilitate subsequent binding to the other binding site on the same CaMKII subunit.

The affinity of calmodulin for the low-affinity binding site was 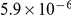 M, corresponding to the low-affinity peptide used by Tse et al. [19]. Meyer et al. have reported a forward rate of 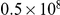 M-1

s-1 for calmodulin binding to a phosphorylated CaMKII dodecamer [18]. To compute a microscopic forward rate for an individual subunit, we divided this number by 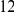 (the number of subunits that make up a holoenzyme) and obtained a rate of 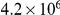 M-1

s-1. This is close to the value of 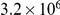 M-1

s-1 reported by Tzortzopoulos and Török [48].

Just like the short fragment used by Tse et al. [19] corresponds to the low-affinity binding site, we took the long fragment (291–312) to correspond to the high-affinity binding site. This is also backed up by earlier findings that have implied residues contained in the longer fragment in high-affinity binding, notably residues 293–295 and residues 296–298 [33]. It has also been observed that binding of calmodulin to a similar fragment (residues 290–314) mimicked calmodulin binding to the entire phosphorylated kinase, suggesting that the entire high-affinity binding domain is indeed contained in this fragment [49]. Since access to the binding site is more constrained in the context of an entire holoenzyme (compared to a relatively short fragment), we expect the in vivo 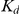 for high-affinity binding of calmodulin to be higher than that. The 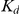 of the long fragment thus provides a lower limit. This 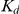 is given by Tse et al. as 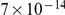 M [19], but Waxham et al. report a 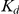 of 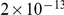 M for the same fragment [33].

Meyer et al. [18] report a 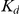 of 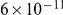 for calmodulin binding to autonomous CaMKII. In our model, autonomous CaMKII will not display subunit opening or closing, so the apparent calmodulin dissociation constant is only a function of the microscopic low-affinity and high-affinity dissociation constants. Since the dissociation constant of the low-affinity binding site, as reported above, is several orders of magnitude bigger than the combined dissociation constant, we concluded that the low-affinity binding site contributes very little to the overall affinity of the fragment and that the low overall 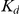 is due almost entirely to the high-affinity binding site. We therefore set the microscopic 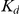 for the high-affinity binding site to 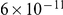 M. This is indeed higher than the 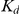 values reported elsewhere for the long fragment [19], [33].

In terms of Gibbs free energies, the 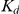 for high-affinity binding we chose corresponds to a (standardised) 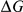 of 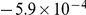 J/mol, and the 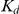 for low-affinity binding to a 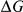 of 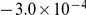 J/mol. Although the number of - partly hidden - assumptions underlying energy calculation makes it difficult to directly compare these values with the energies reported for the protein complex in the molecular dynamics simulations, in both cases the ratio between the high-affinity and the low-affinity energy is similar.

We assumed that “sliding” of calmodulin from the low-affinity site to the high-affinity site is intrinsically symmetrical, i. e. that once dissociated from one binding site, the probability of binding to the other binding site should be the same in either direction. Therefore, the equilibrium constant for the sliding reaction (and hence, the probability of sliding from one site to the other) should be determined by the dissociation rates (and hence, the dissociation constants) for both sites:
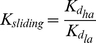
(1)


Using this equation, we concluded that calmodulin bound to the low-affinity site will “slide” to the high-affinity binding site with a probability of 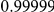.

### Subunit opening and closing

In order to obtain an estimate of the opening probability of a single CaMKII subunit, we used COPASI [50] and constructed a model of calmodulin binding to a single CaMKII subunit which could open or close based on the above parameters. The model contains only ten reactions, six of which describe binding and dissociation of calmodulin to the two binding sites on an unoccupied CaMKII subunit (in the open state for the high-affinity binding site, in both the open and the closed state for the low-affinity binding site), two describe “sliding” of calmodulin between the two binding sites and two describe opening and closing of the CaMKII subunit. The full list of reactions is given in Table S1.

The calmodulin concentration used in this model was 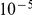 M and the concentration of CaMKII peptide was 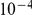 M, corresponding to the experimental concentrations used by Tse et al. [19]. We started the simulations with all of the peptide in the unbound state.

This model was used to optimise the probability of CaMKII opening. As an objective function, we used the apparent combined 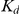 for a single subunit:
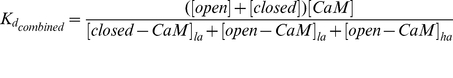
(2)


This value has been reported by Tzortzopoulos and Török [48] to be 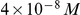 for calmodulin binding to CaMKII with a T286A mutation. This was chosen in order to isolate the effects of calmodulin binding and opening/closing only, without having to account for autophosphorylation. The *Genetic Algorithm SR* optimisation function in COPASI [50] was run ten times, with ten randomly chosen initial values. We used the default settings for the *Genetic Algorithm SR* function (200 generations, a population size of 20 and random number generator 1, all other values set to zero). The resulting value for the opening probability was 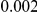.

All other parameters were taken from the literature. A full list is given in table 2.

**Table 2:** List of parameters for the model of calmodulin trapping.

Parameter	value	reference
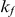 for CaMKII phosphorylation at residue 286	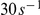	[55]
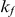 for dephosphorylation of CaMKII at residue 286 by PP1	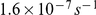	computed from [56]
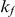 for CaMKII phosphorylation at residue 306	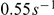	[55]
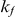 for dephosphorylation of CaMKII at residue 306 by PP1	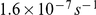	computed from [56]
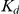 for calmodulin binding to the low-affinity site of CaMKII	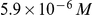	[19]
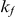 for calmodulin binding to the low-affinity site of CaMKII	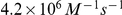	[18]
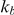 for calmodulin binding to the low-affinity site of CaMKII	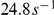	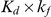
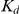 for calmodulin binding to the high-affinity site of CaMKII	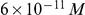	*this study*
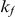 for calmodulin binding to the high-affinity site of CaMKII	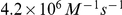	[18]
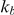 for calmodulin binding to the high-affinity site of CaMKII	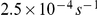	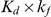
probability of spontaneous CaMKII opening	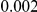	*this study*
probability of calmodulin sliding to the high-affinity site	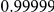	*this study*
total number of CaMKII subunits used for simulation	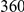	total number of CaMKII subunits in the PSD [52]
total number of calmodulin molecules used for simulation	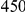	*this study*
reaction volume used for simulation	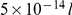	*this study*

List of parameters for the model of calmodulin trapping.

### Stochastic simulations

Stochastic simulations were performed using StochSim [51]. The total duration of the simulation was 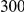 s.The software was allowed to optimise the time increment, with data being read out every millisecond. We used a total of 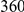 CaMKII subunits, corresponding to the number of CaMKII subunits typically found in a postsynaptic density [52], in order to facilitate future simulations under physiological conditions. Both the number of calmodulin molecules (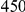) and the total reaction volume (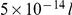) were chosen to preserve the concentrations and CaMKII-to-calmodulin ratio used in the experiments performed by Meyer et al. [18].

For the two-binding-site wildtype model, all CaMKII subunits were closed, unphosphorylated and not bound to calmodulin at the outset of the simulation. Calmodulin was present at the beginning, but removed after 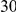 s to mimic calcium withdrawal. The complete StochSim input files for this model are given in Dataset S3. The T286A mutation was implemented by setting the Thr286 autophosphorylation rate to zero in a model otherwise identical to that for the wildtype.

The one-binding-site model was the same as the two-binding-site model, except that the reaction describing calmodulin binding to the low-affinity site and the rapid equilibrium governing calmodulin “sliding” from the low-affinity to the high-affinity site were removed. Everything else (including the definition of the state flags) was kept as it was. In the one-binding site scenario, Thr286 phosphorylation also had a marked effect on the apparent on rate of calmodulin binding. In order to separate this effect from the actual trapping effect (the change in off rate), the initial calmodulin binding phase was not explicitly modelled. Instead, all 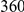 CaMKII subunits were open, calmodulin-bound and (for wildtype CaMKII) phosphorylated at Thr286 at the outset of the simulation, and calmodulin was withdrawn from the beginning of the simulation. Simulations were run for 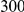 s, with an automatically optimised time step, as above. Again, the T286A mutant differed from the wildtype by the Thr286 autophosphorylation rate being set to zero.

To allow for direct comparison between the two-binding-site and one-binding-site models, simulations of the two-binding-site models were run under the same conditions as for the one-binding-site model, i. e. with all CaMKII calmodulin-bound at the outset and calmodulin withdrawn at the beginning of the simulation.

Ten StochSim simulation runs were performed for each of the models. All simulations were run on a Centos 5.4 Linux LSF Cluster containing 350 nodes with 32GB RAM or more each. The longest simulations took a few hours to complete.

## Supporting Information

Table S1 **We used a simple model of a single CaMKII subunit which could open, close and bind to calmodulin to determine the opening probability of CaMKII using the parameter search facility of COPASI** [**50**] **.** The full list of reactions of this model is given in this table.
(PDF)

Dataset S1 **Structural model of calmodulin binding to the low-affinity binding site on a closed subunit of CaMKII.**
(TXT)

Dataset S2 **Structural model of calmodulin binding to the high-affinity binding site on a CaMKII subunit.** When the inhibitory helix of CaMKII is allowed some flexibility, it moves away from the catalytic domain to accommodate for calmodulin binding to the high-affinity binding site. This exposes the catalytic site and the Thr286 autophosphorylation site.
(TXT)

Dataset S3 **This dataset is a text file containing the input files for simulation with StochSim.** Different parts of the model and the simulation are defined in section that must be copied to different files, as follows: STCHSTC.INI: Controls the parameters of the simulation, such as the time interval, the total simulation duration and the names of input and output files. MESSAGE.INI: List of StochSim error messages for troubleshooting should the simulation exit. COMPLEX.INI: Contains information about the different components of the model and their initial concentrations. REACTION.INI: Contains all reactions, specifying substrates, products, and forward and backward reaction rates. MS_1.INI: Contains information pertaining to the state flags of CaMKII and to how these are affected by reactions and rapid equilibria. NS_1.INI Contains information about neighbour-sensitive reactions (in this case, only Thr286 phosphorylation). ARRAY.INI: Defines the geometry and composition of arrays (in this case, 60 hexamers of CaMKII). DYNAMIC.INI: Contains information about parameter values that change over time, used here to set the concentration of active calmodulin to zero after 30 seconds.
(TXT)
