# Supplementary material for: Structural Analysis and Stochastic Modelling Suggest a Mechanism for Calmodulin Trapping by CaMKII
Source: PLoS One. 2012 Jan 18;7(1):e29406. doi: 10.1371/journal.pone.0029406 (PMC3261145; doi:10.1371/journal.pone.0029406)
Supplement: Table S1 — We used a simple model of a single CaMKII subunit which could open, close and bind to calmodulin to determine the opening probability of CaMKII using the parameter search facility of COPASI [50] . The full list of reactions of this model is given in this table. (PDF) [file pone.0029406.s001.pdf]

Table S3: **List of reactions for small model of calmodulin binding and subunit opening to determine opening probability.**

$$
\begin{aligned}
\text{calm} + \text{open} &\rightarrow \text{open-la} \\
\text{open-la} &\rightarrow \text{calm} + \text{open} \\
\text{calm} + \text{closed} &\rightarrow \text{closed-la} \\
\text{closed-la} &\rightarrow \text{calm} + \text{closed} \\
\text{calm} + \text{open} &\rightarrow \text{open-ha} \\
\text{open-ha} &\rightarrow \text{calm} + \text{open} \\
\text{open-la} &\rightarrow \text{open-ha} \\
\text{open-ha} &\rightarrow \text{open-la} \\
\text{open} &\rightarrow \text{closed} \\
\text{closed} &\rightarrow \text{open}
\end{aligned}
$$

List of reactions for small model of calmodulin binding and subunit opening to determine opening probability. Here, “calm” denotes calmodulin, “closed” the closed form of a CaMKII subunit, “open” the open form, and “open-la”, “closed-la” and “open-ha” denote the open or closed for of CaMKII bound to calmodulin at the low-affinity or high-affinity binding site, respectively.
